# Brain metastases at the time of presentation of non-small cell lung cancer: a multi-centric AERIO[Fn tlnote1] analysis of prognostic factors

**DOI:** 10.1054/bjoc.2000.1706

**Published:** 2001-04

**Authors:** W Jacot, X Quantin, J-M Boher, F Andre, L Moreau, M Gainet, A Depierre, E Quoix, T Le Chevalier, J-L Pujol

**Affiliations:** Department of Chest Diseases, Hôpital Universitaire Arnaud de Villeneuve, 34295 Montpellier Cedex 5, France; Department of Statistics and Epidemiology, University Institute for Clinical Research, Hôpital Universitaire Arnaud de Villeneuve; Cancer institute. Institute Gustave Roussy, Villejuif, France; Department of Chest Diseases, Hôpital Universitaire de Strasbourg, France; Department of Chest Diseases, Hôpital Universitaire de Besançon, France

**Keywords:** brain metastases, non-small cell lung cancer, neuron-specific enolase, prognosis

## Abstract

A multi-centre retrospective study involving 4 French university institutions has been conducted in order to identify routine pre-therapeutic prognostic factors of survival in patients with previously untreated non-small cell lung cancer and brain metastases at the time of presentation. A total of 231 patients were recorded regarding their clinical, radiological and biological characteristics at presentation. The accrual period was January 1991 to December 1998. Prognosis was analysed using both univariate and multivariate (Cox model) statistics. The median survival of the whole population was 28 weeks. Univariate analysis (log-rank), showed that patients affected by one of the following characteristics proved to have a shorter survival in comparison with the opposite status of each variable: male gender, age over 63 years, poor performance status, neurological symptoms, serum neuron-specific enolase (NSE) level higher than 12.5 ng ml^−1^, high serum alkaline phosphatase level, high serum LDH level and serum sodium level below 132 mmol l^−1^. In the Cox's model, the following variables were independent determinants of a poor outcome: male gender: hazard ratio (95% confidence interval): 2.29 (1.26–4.16), poor performance status: 1.73 (1.15–2.62), age: 1.02 (1.003–1.043), a high serum NSE level: 1.72 (1.11–2.68), neurological symptoms: 1.63 (1.05–2.54), and a low serum sodium level: 2.99 (1.17–7.62). Apart from 4 prognostic factors shared in common with other stage IV NSCLC patients, whatever the metastatic site (namely sex, age, gender, performance status and serum sodium level) this study discloses 2 determinants specifically resulting from brain metastasis: i.e. the presence of neurological symptoms and a high serum NSE level. The latter factor could be in relationship with the extent of normal brain tissue damage caused by the tumour as has been demonstrated after strokes. Additionally, the observation of a high NSE level as a prognostic determinant in NSCLC might reflect tumour heterogeneity and understimated neuroendocrine differentiation. © 2001 Cancer Research Campaignhttp://www.bjcancer.com


*British Journal of Cancer* (2001) **84**(7), 903–909
© 2001 Cancer Research Campaign
doi: 10.1054/ bjoc.2000.1706, available online at http://www.idealibrary.com on IDEAL®

http://www.bjcancer.com

### Brain metastases at the time of presentation of non-small cell lung cancer: a multi-centric AERIO* analysis of prognostic factors

**W Jacot[1], X Quantin[1], J-M Boher[2], F Andre[3], L Moreau[4], M Gainet[5], A Depierre[5], E Quoix[4], T Le Chevalier[3] and J-L Pujol[1,2]**

Department of Chest Diseases, Hôpital Universitaire Arnaud de Villeneuve, 34295 Montpellier Cedex 5, France; [2]Department of Statistics and Epidemiology, University Institute for Clinical Research, Hôpital Universitaire Arnaud de Villeneuve; [3]Cancer institute. Institute Gustave Roussy, Villejuif, France; [4]Department of Chest Diseases, Hôpital Universitaire de Strasbourg, France; [5]Department of Chest Diseases, Hôpital Universitaire de Besançon, France

**Summary** A multi-centre retrospective study involving 4 French university institutions has been conducted in order to identify routine pre-therapeutic prognostic factors of survival in patients with previously untreated non-small cell lung cancer and brain metastases at the time of presentation. A total of 231 patients were recorded regarding their clinical, radiological and biological characteristics at presentation. The accrual period was January 1991 to December 1998. Prognosis was analysed using both univariate and multivariate (Cox model) statistics. The median survival of the whole population was 28 weeks. Univariate analysis (log-rank), showed that patients affected by one of the following characteristics proved to have a shorter survival in comparison with the opposite status of each variable: male gender, age over 63 years, poor performance status, neurological symptoms, serum neuron-specific enolase (NSE) level higher than 12.5 ng $ml^{-1}$, high serum alkaline phosphatase level, high serum LDH level and serum sodium level below 132 mmol $l^{-1}$. In the Cox's model, the following variables were independent determinants of a poor outcome: male gender: hazard ratio (95% confidence interval): 2.29 (1.26–4.16), poor performance status: 1.73 (1.15–2.62), age: 1.02 (1.003–1.043), a high serum NSE level: 1.72 (1.11–2.68), neurological symptoms: 1.63 (1.05–2.54), and a low serum sodium level: 2.99 (1.17–7.62). Apart from 4 prognostic factors shared in common with other stage IV NSCLC patients, whatever the metastatic site (namely sex, age, gender, performance status and serum sodium level) this study discloses 2 determinants specifically resulting from brain metastasis: i.e. the presence of neurological symptoms and a high serum NSE level. The latter factor could be in relationship with the extent of normal brain tissue damage caused by the tumour as has been demonstrated after strokes. Additionally, the observation of a high NSE level as a prognostic determinant in NSCLC might reflect tumour heterogeneity and understimated neuroendocrine differentiation. © 2001 Cancer Research Campaign http://www.bjcancer.com

**Keywords:** brain metastases; non-small cell lung cancer; neuron-specific enolase; prognosis

Patients with lung cancer frequently suffer from brain metastases at the time of presentation. This condition affects approximately 10% of non-small cell lung cancer (NSCLC) patients (Newman and Hansen, 1974; Sorensen et al, 1988). Surgery is feasible only for a small proportion of these patients. Whole brain radiotherapy has been, hitherto, the generally recommended treatment in inoperable patients. The survival of NSCLC patients with brain metastases is poor, reported to be between 3 to 6 months in patients treated with medical therapies, either radiotherapy or chemotherapy (compared to 6–10 months in other advanced NSCLC (Paesmans et al, 1995; Shepherd, 1999)). Furthermore, brain metastases at the time of presentation of lung cancer seems to be a worse prognosis (Sorensen et al, 1988) than metachronous brain metastases.

New therapeutic strategies are needed to improve the outcome of these patients. The knowledge of prognostic determinants might be important in both clinical trials and routine practice (Komaki et al, 1993; Charloux et al, 1997; Paesmans et al, 1997; Merrill et al, 1999). In the former setting, prognostic co-variables must be taken into account in survival analyses; by way of illustration, in a given randomized trial, the statement that a difference in survival is related to the effects of the treatment must be supported by a proportional hazards model demonstrating that this effect does not depend on well-known prognostic determinants (Depierre et al, 1999; Furuse et al, 1999). In the second setting, a therapeutic decision might be influenced by the state of prognostic variables (Komaki et al, 1993).

Here we especially take a look at the prognostic significance of 2 specific serum markers, CYFRA 21-1 and neuron-specific enolase (NSE). The prognostic value of CYFRA 21-1 (a fragment of cytokeratin subunit 19) in this disease has been suggested (Pujol et al, 1993; Wieskopf et al, 1995; Brechot et al, 1997). NSE, the γ-subunit of enolase, has been widely investigated as a marker of small cell lung cancer (SCLC; Jorgensen et al, 1989). Although only a small proportion of NSCLC presented with a high NSE level, this marker might indirectly reflect; i) a neuroendocrine component of the disease in favour of tumour heterogeneity; ii) a

*Received 25 July 2000*
*Revised 13 December 2000*
*Accepted 21 December 2000*

*Correspondence to:* J-L Pujol

* "Association d'Enseignement et de Recherche des Internes en Oncologie" (residents in oncology association for education and research), 36 rue des Vinaigriers, 75010 Paris, France, Fax 33 1 42 05 81 44

marker of brain damage. In order to evaluate prognostic variables in NSCLC patients with brain metastases at the time of presentation, we conducted a retrospective study.

### PATIENTS AND METHODS

### Patient selection

This is a multi-centre retrospective study involving 4 French university institutions (Montpellier university hospital, Institut Gustave Roussy, Strasbourg university hospital and Besançon university hospital). In the past, these institutions were involved in numerous cancer trials and therefore they possess comprehensive patient databases. Case reports extracted from these databases were selected on the following criteria: histologically proven NSCLC, brain metastasis at the time of presentation as demonstrated either by computed tomography (CT) or magnetic resonance imaging (MRI), no prior anti-cancer therapy. The accrual period was January 1991 to December 1998. Histological sub-classification was done according to the WHO classification (World Health Organization, 1982). Staging was carried out by exhaustive procedures according to the 4th edition of the Union Internationale Contre le Cancer (UICC) tumour node metastases (TNM) classification (Sobin et al, 1987) and the American Thoracic Society map of regional pulmonary nodes (Tisi et al, 1982). By definition, all patients belonged to stage IV of the new Mountain's stage grouping (Mountain, 1997)

### Data collection

For each patient, the following pre-treatment characteristics were recorded: age, sex, performance status (estimated according to the Eastern Cooperative Oncology Group (Zubrod et al, 1960)), percentage of weight loss during the previous 4 months, tumour and nodal status, histology, clinical symptoms belonging to brain metastases (i.e. intra-cranial hypertension, seizure, focal neurological symptoms), other metastatic sites involved (i.e. liver, adrenal glands, bone metastases), serum CYFRA 21-1 level (upper limit of normal values: 3.6 ng $ml^{-1}$), serum NSE level (upper limit of normal values: 12.5 ng $ml^{-1}$), serum alkaline phosphatase and lactate dehydrogenase (LDH) levels (either normal or elevated, depending on the institution's upper normal values), serum sodium level (lower normal limit 132 mmol $l^{-1}$), white blood cell count, serum albumin level, number and location of brain metastases on CT or MRI and finally, treatment modalities.

### Statistics

Survival data were updated on February 1 1999. Survival was defined as the time from histological diagnosis to the date of death. Death related to the disease whichever the progression site, or related to its treatment was analysed as an event. Deaths from other causes were treated as censored observations (myocardial infarctions or pulmonary embolisms). Survival was estimated by the Kaplan–Meier method (Kaplan and Meier, 1958). Single variable survival analyses were done by means of log-rank tests.

Coding methods for the different variables depended on their nature. Some of the variables have been extensively described in the literature therefore the threshold has been defined from previous publications. Performance status has been analysed according to 2 classical modalities: PS 0–1 and PS greater or equal to 2 (Zubrod et al, 1960). The effect of nodal status on prognosis was tested according to the presence or the absence of mediastinal lymph node involvement. The same coding regarding tumour status has been adopted according to the new Mountain's stage grouping (Mountain, 1997). Regarding biological variables, including tumour markers we used previously published thresholds: 3.6 ng $ml^{-1}$ for CYFRA 21-1 (Pujol et al, 1993). The threshold values for serum NSE levels to be used in clinical studies have been defined from publications describing this neuroendocrine marker (Cooper and Splinter, 1987; Jorgensen et al, 1989). The treatment modality was not tested as a prognostic variable inasmuch as treatment was decided according to each institution's procedure and was based upon the different pre-treatment variables.

Multivariate regression was done with the Cox model (Cox, 1972; Andersen, 1991). The forward selection of variable procedure has been used. The selection of variables to be tested in the Cox model was made using the results of univariate analysis i.e. variables reaching at least a *P* level less than 15%. This model was written after a binary coding of the significant variables (except for age which was analysed as a continuous variable): categorical variables (such as performance status) were transformed into binary variables (0: negative or 1: positive). The number of levels of a categorical variable needed to describe a predictive factor is one less than the categories of that factor inasmuch as its baseline level is defined by setting the value of each of the categorical variables at zero. The significance of the effect of a given factor was assessed by determining whether or not the coefficient assigned to one or more of its categories was sufficiently different from zero. The proportional hazard assumption for each of the selected variables retained in the final model was originally checked by plotting the log cumulative baseline hazard ratio. A *P* level of less than 0.05 was considered significant. SAS software package was used.

According to the above-mentioned procedure, 14 variables were selected as putative prognostic determinants to be tested in the Cox regression hazard model. They represented less than 10% of the total of observed events (207 deaths) and therefore complied with the current recommendation (Harrell et al, 1985).

### RESULTS

Patient's characteristics are summarized in Table 1. Most of the main characteristics of NSCLC were retrieved particularly a median age of 59 years (range, 32–85 years). 85 patients (37%) did not have symptoms related to the brain metastases and the disease was disclosed by a pre-treatment staging procedure including CT scan. 134 patients suffered from neurological symptoms, consisting of intra-cranial hypertension symptoms (33 patients), seizure, epilepsy or muscle weakness (101 patients) or an association of these different symptoms. There were 6 deaths related neither to the disease nor to the treatment. These observations have been censored. At the time of analysis, 207 deaths had been reported and 24 (10%) patients were still alive. In the whole patient population, median survival was 28 weeks (95% confidence interval [CI], 24 to 34 weeks). The 1- and 2-year survival rates were 25% (95% CI, 19–31%) and 8% (95% CI, 4–11%), respectively (Figure 1).

### Univariate analysis

Univariate analysis (Table 2) showed that patients affected by one of the following characteristics proved to have a shorter survival in

© 2001 Cancer Research Campaign

**Table 1** Patients' characteristics

| Variables | | No. of patients (%) |
|---|---|---|
| Total | | 231 |
| Age (years) | Median ± SD | 59 ± 11 |
| | < 40 | 13 (6) |
| | 40–49 | 49 (21) |
| | 50–59 | 65 (28) |
| | 60–69 | 70 (30) |
| | 70 and over | 34 (15) |
| Male gender | Male | 194 (84) |
| ECOG performance status | 0 | 44 (19) |
| | 1 | 92 (40) |
| | 2 | 60 (26) |
| | 3 | 26 (11) |
| | 4 | 9 (4) |
| Tumour status | 1–2 | 107 (47) |
| | 3–4 | 122 (53) |
| Nodal status | 0–1 | 64 (28) |
| | 2–3 | 165 (71) |
| Histology | Squamous cell carcinoma | 95 (41) |
| | Adenocarcinoma | 86 (37) |
| | Large cell carcinoma | 50 (22) |
| Weight loss (%) | < 5% /≥ 5% | 119 (52)/88 (38) |
| | Unknown | 24 (10) |
| Serum Cyfra 21–1 level | < 3.6 /≥ 3.6 | 58 (25)/88 (38) |
| | Unknown | 85 (37) |
| Serum NSE level | ≤ 12.5 /> 12.5 | 142 (61)/57 (25) |
| | Unknown | 32 (14) |
| Serum albumin level | <32 g $l^{-1}$ /≥ 32 g $l^{-1}$ | 159 (69)/27 (12) |
| | Unknown | 45 (19) |
| Serum sodium level | <132 mmol $l^{-1}$ /≥ 132 mmol $l^{-1}$ | 219 (95)/10 (4) |
| | Unknown | 2 (1) |
| Alkaline phosphatase | Normal/elevated | 180 (78)/41 (18) |
| | Unknown | 10 (4) |
| Lactate dehydrogenase level | Normal/elevated | 133 (58)/78 (34) |
| | Unknown | 20 (9) |
| Blood leucocyte count | ≤ 10 000 $\mu l^{-1}$> 10 000 µl | 108 (47)/120 (52) |
| | Unknown | 3 (1) |
| Adrenal gland metastases | Yes/No | 34 (15)/197 (85) |
| Bone metastases | Yes/No | 47 (20)/184 (80) |
| Liver metastases | Yes/No | 24 (10)/207 (90) |
| No. of brain metastases | Unique/Multiples | 89 (39)/125 (54) |
| | Unknown | 17 (7) |
| Site of brain metastases | Supra/Infra-tentorial | 144 (62) / 19 (8) |
| | Mixed | 49 (21) |
| | Unknown | 19 (8) |
| Neurologic symptoms | No/Yes | 85 (37)/134 (58) |
| Treatment modalities | Best Supportive Care | 19 (8%) |
| | Radiotherapy | 13 (6%) |
| | Chemotherapy | 41 (18%) |
| | Surgery | 0 |
| | Surgery + Chemotherapy | 2 (1%) |
| | Surgery + Radiotherapy | 2 (1%) |
| | Surgery + Chemo. + Radio. | 3 (1%) |
| | Radiotherapy + Chemotherapy | 150 (65%) |
| Brain response to treatment | Yes/No | 93 (40)/107 (46) |
| | Unknown | 31 (13) |

comparison with the opposite status of each variable: male gender, age over 63 years, performance status equal to or worse than 2, neurological symptoms (Figure 2), serum NSE level higher than 12.5 ng $ml^{-1}$ (Figure 3), high serum alkaline phosphatase level, high serum LDH level and serum sodium level lower than 132 mmol $l^{-1}$.

*© 2001 Cancer Research Campaign*

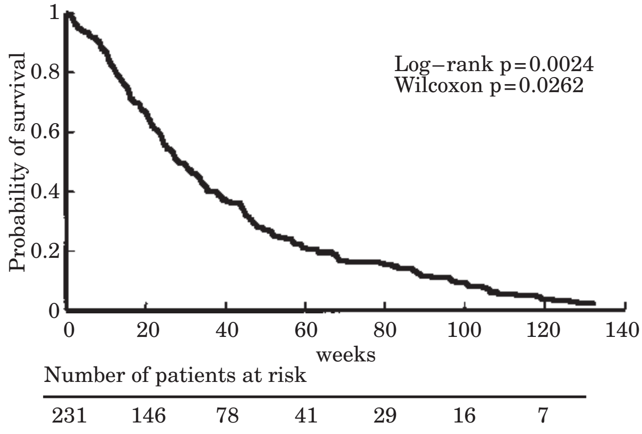

**Figure 1** Kaplan–Meier estimation of overall survival in the whole population of non-small cell lung cancer patients suffering from brain metastases at the time of presentation

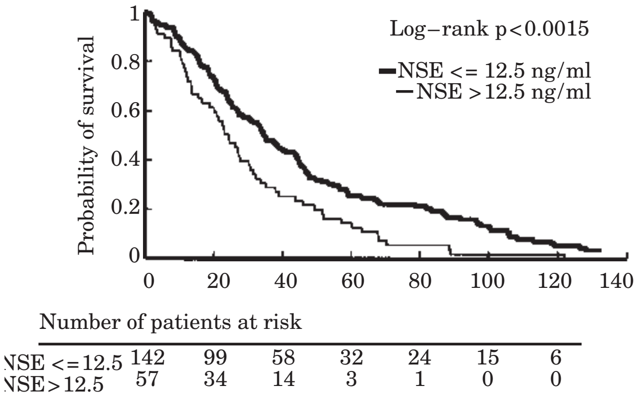

**Figure 2** Probability of survival of non-small cell lung cancer patients with or without neurological symptoms

### Multivariate analysis

According to the above-mentioned procedure, 14 variables were selected as putative prognostic determinants to be tested in the Cox regression hazard model (sex, age, performance status, histology, serum NSE level, serum CYFRA 21-1 level, serum albumin, alkaline phosphatases, LDH, serum sodium, blood leucocyte count, presence of bone metastases, presence of liver metastases, neurological symptoms). They represented less than 10% of the total observed events (207 deaths) and therefore complied with the current recommendation (Harrell et al, 1985).

The following variables were independent determinants of a poor outcome: male gender: hazard ratio (95% confidence interval): 2.29 (1.26–4.16), poor performance status: 1.73 (1.15–2.62), age: 1.02 (1.003–1.043), a high serum NSE level: 1.72 (1.11–2.68), neurological symptoms: 1.63 (1.05–2.54), and a low serum sodium level: 2.99 (1.17–7.62) (Table 3).

Finally, patients have been coded according to the presence or absence of a major metastatic site (i.e. presence of at least one of the following metastatic sites: liver or adrenal or bone). This variable did not modify the results of the Cox model.

### DISCUSSION

Brain metastases at the time of presentation of NSCLC are a frequent clinical problem. Classically, treatment consists of whole brain radiotherapy. Surgery is usually proposed to the small subset of patients presenting with a single brain metastasis and for whom primary site can be controlled. The role of chemotherapy in the management of NSCLC with brain involvement remains controversial. Short life expectancy is generally considered as a deterrent to curative intent. However, recent studies indicate that chemotherapy is active on brain metastases of NSCLC (Ellis et al, 1998; Kelly and Bunn, 1998; Postmus and Smit, 1999). In addition, new therapies such as radiosurgery and combined chemotherapy-radiotherapy are being developed for these patients. Therefore, the appraisal of the prognostic factor is mandatory.

We report herein a survival analysis of a homogeneous population of NSCLC patients with brain metastases at the time of presentation. 4 prognostic factors elicited from this study are classical survival determinants reported to be shared in common by all NSCLC whatever the metastatic site (Zimm et al, 1981; Diener-West et al, 1989; Komarnicky et al, 1991; Lonjon et al, 1994; Ryan et al, 1995; Ando et al, 1996; Auchter et al, 1996; Gaspar et al, 1997; Hsiung et al, 1998; Agboola et al, 1998; Lagerwaard et al, 1999), or by brain metastases whatever the primary tumour (Zimm et al, 1981; Diener-West et al, 1989; Komarnicky et al, 1991; Lonjon et al, 1994; Auchter et al, 1996; Gaspar et al, 1997; Agboola et al, 1998; Lagerwaard et al, 1999). These factors are gender, performance status, age and serum sodium level.

Apart from the above-mentioned factors, our study disclosed 2 determinants which might result from brain metastases: the presence of neurological symptoms and a high serum NSE level. Clinical symptoms related to the brain metastases were the only site-specific factor independently affecting survival. Neither the number nor the location of brain metastases were statistically significant determinants of prognosis. This finding contrasts with some other studies also aimed at prognosticating the outcome of patients suffering from brain metastases (Zimm et al, 1981; Swift et al, 1993; Nussbaum et al, 1996; Sen et al, 1998). However, one can mention that these determinants vary from one study to another (Zimm et al, 1981; Swift et al, 1993; Ando et al, 1996; Nussbaum et al, 1996; Hsiung et al, 1998; Nguyen et al, 1998; Sen et al, 1998). This discrepancy could be in relationship with a possible underestimation of the number of metastases and the tumour burden shown by means of CT scan. Therefore, the case of anatomic characteristics of brain metastases seems of less prognostic importance than the presence of symptoms by themselves. This statement does not minimize the paramount consequence of anatomic characterization of brain disease in treatment decision.

The $\gamma\gamma$ isomer of the ubiquitous enzyme enolase referred to as NSE is the most widely used neuroendocrine serum marker in SCLC clinical management (Cooper and Splinter, 1987; Jorgensen et al, 1989). In the NSCLC histology, the evaluation of this neuroendocrine marker might seem unexpected. However, the common endodermal origin of all histological types of lung cancer makes it possible to include SCLC and NSCLC in a unique spectrum of differentiation with frequent overlaps (Yesner and Carter, 1982). Early studies using histology (Yesner and Carter, 1982) or electronic microscopy (Gould et al, 1983) have demonstrated that mixed SCLC-NSCLC may be observed in a low proportion of all lung cancers. Patients with mixed SCLC-large cell carcinoma proved to have a shorter survival than those with pure histological

 *© 2001 Cancer Research Campaign*

**Table 2** Univariate analysis

| Variable | | Median survival (weeks) | *P* (Log- rank) |
|---|---|---|---|
| Age (year) | ≤ 63 | 33.7 | 0.0363 |
| | > 63 | 21.4 | |
| Gender | Female | 50.7 | 0.0004 |
| | Male | 26.3 | |
| ECOG performance status | < 2 | 35.3 | 0.0003 |
| | ≥ 2 | 20.9 | |
| Tumour status (T) | 1–2 | 30.6 | 0.2923 |
| | 3–4 | 26.9 | |
| Nodal status (N) | 0–1 | 27.3 | 0.2247 |
| | 2–3 | 29.6 | |
| Histology | Squamous-cell carcinoma | 26.3 | 0.1075 |
| | Adenocarcinoma | 32.7 | |
| | Large cell carcinoma | 28.4 | |
| Weight loss | < 5% | 28.4 | 0.4312 |
| | ≥ 5% | 27.3 | |
| Serum Cyfra 21–1 level | ≤ 3.6 | 33.6 | 0.1314 |
| | > 3.6 | 24.6 | |
| Serum NSE level | ≤ 12.5 | 34.4 | 0.0015 |
| | > 12.5 | 24.3 | |
| Serum albumin level | < 32 g $l^{-1}$ | 20.1 | 0.1293 |
| | ≥ 32 g $l^{-1}$ | 31.4 | |
| Serum sodium level | < 132 | 15.4 | 0.0141 |
| | ≥ 132 | 30.1 | |
| Serum alkaline phosphatase level | Normal | 32.3 | 0.0080 |
| | Elevated | 20.6 | |
| Serum lactate dehydrogenase level | Normal | 33.6 | 0.0358 |
| | Elevated | 23.6 | |
| Blood leukocyte count | ≤ $10.10^9$ $l^{-1}$ | 33.6 | 0.1005 |
| | > $10.10^9$ $l^{-1}$ | 27 | |
| Adrenal gland metastases | Yes | 24.3 | 0.4637 |
| | No | 29.6 | |
| Bone metastases | Yes | 23.9 | 0.0663 |
| | No | 30.3 | |
| Liver metastases | Yes | 24.1 | 0.1414 |
| | No | 28.4 | |
| No. of brain metastases | Unique | 30.6 | 0.1675 |
| | Multiples | 28 | |
| Site of brain metastases | Supra-tentorial | 32.3 | 0.7317 |
| | Infra-tentorial | 27.3 | |
| | Mixed | 26.9 | |
| Neurologic symptoms | No symptoms | 38.7 | 0.0019 |
| | Neurologic symptoms | 24.3 | |

SCLC suggesting that this heterogeneity has clinical relevance (Radice et al, 1982). Therefore, we decided to evaluate this marker in the particular setting of brain metastasis of NSCLC.

**Table 3** Estimated hazard ratio for significant variables

| Variables | Hazard ratio | 95% CI | *P* |
|---|---|---|---|
| Male gender | 2.29 | 1.26–4.16 | 0.006 |
| Poor performance status (2–4) | 1.73 | 1.15–2.62 | 0.009 |
| Age | 1.02 | 1.003–1.043 | 0.021 |
| High serum NSE level | 1.72 | 1.11–2.68 | 0.016 |
| Presence of neurological symptoms | 1.63 | 1.05–2.54 | 0.026 |
| Low serum sodium level | 2.99 | 1.17–7.62 | 0.022 |

In our study patients with a pre-treatment high serum NSE level proved to have a poor outcome. Two hypotheses could explain this finding and they are not mutually exclusive. First, this high NSE level might reflect a neuroendocrine differentiation. This heterotopic antigen expression could be regarded as a consequence of a phenotypic heterogeneity, a unique characteristic of human malignancy thought to be in relationship with genotypic instability and tumour progression (Nicolson, 1987). Alternatively, high serum NSE levels may reflect the extent of the neuronal damage. One piece of evidence which can support this hypothesis is the relationship between the degree of neuronal damage and the serum NSE level following a cerebral stroke (Cunningham et al, 1991, 1996; DeGiorgio et al, 1995, 1999; Fogel et al, 1997; Missler et al, 1997; Martens et al, 1998; Buttner et al, 1999; Schoerkhuber et al, 1999; Wunderlich et al, 1999) or other neuronal brain damage (DeGiorgio et al, 1995, 1999; Fogel et al, 1997; Martens et al,

*© 2001 Cancer Research Campaign* 

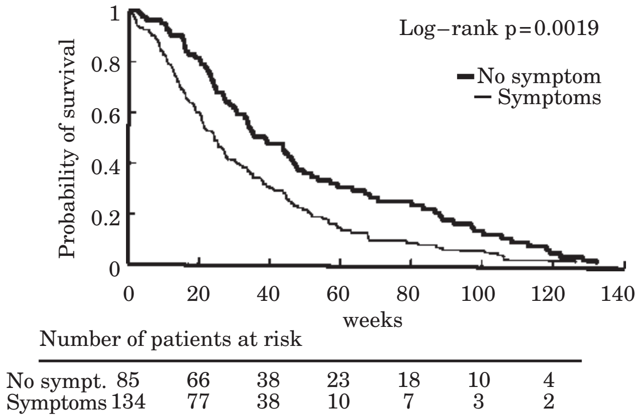

**Figure 3** Probability of survival of non-small cell lung cancer patients with normal and elevated pre-treatment serum NSE level

1998; Buttner et al, 1999; Schoerkhuber et al, 1999). In these diseases, the serum NSE level exhibits a prognostic indication inasmuch as studies have found a close relationship between the volume of affected neuronal tissue and the serum NSE level (Cunningham et al, 1991, 1996; Missler et al, 1997; Wunderlich et al, 1999). We hypothesize that the poor outcome of patients with a high NSE level and brain metastasis is due to the severity of normal neuronal tissue damage surrounding metastases. However, the latter explanation and the first hypothesis, i.e. serum NSE as a marker of phenotypic heterogeneity, are not mutually exclusive.

One may hypothesize that, due to the retrospective nature of the herein study, a possible bias was introduced by the treatment heterogeneity (as shown in Table 1). Patients received a combination of chemotherapy or chemo-radiotherapy according to each centre's policy. Each indication was based upon specific variables such as solitary or multiple brain metastases, performance status, etc. This treatment heterogeneity mainly reflects the lack of consensus regarding the management of NSCLC patients affected by brain metastases at time of presentation. There is no clear demonstration in the literature that a given drug combination or a given combined modality could be considered as a standard regimen. In addition, in our population, the distribution of serum NSE levels did not differ according to treatment modality suggesting that the prognostic significance of the marker was not affected by therapy.

The present manuscript reports an exploratory multi-centre study with identification of prognostic determinants taken as the primary endpoint. Therefore, it would be hazardous to draw specific treatment recommendations from our data. According to the classification proposed by Simon and Altman (1994), the study herein could be considered as a type 2 prognostic factor investigation. The findings of the current study deserve further confirmatory investigations in a larger population of patients with homogeneous therapeutic strategies. Such phase III studies are considered as the only means 'to determine which subsets of patients benefit from a given therapy'.

In conclusion, our study confirms age, sex and performance status as prognostic factors of NSCLC with brain metastases at the time of presentation suggesting that this subset of patients shares similar determinants of outcome with the general NSCLC population. In addition, both neurological symptoms and serum NSE levels are site-specific predictors of outcome to be taken into account in new therapeutic approaches in this setting.

### ACKNOWLEDGEMENTS

The authors wish to thank Mrs Jo Baïssus for help in preparing the manuscript.

### REFERENCES

Agboola O, Benoit B, Cross P, Da Silva V, Esche B, Lesiuk H and Gonsalves C (1998) Prognostic factors derived from recursive partition analysis (RPA) of Radiation Therapy Oncology Group (RTOG) brain metastases trials applied to surgically resected and irradiated brain metastatic cases [see comments]. *Int J Radiat Oncol Biol Phys* **42**: 155–159

Andersen PK (1991) Survival analysis 1982–1991: the second decade of the proportional hazards regression model. *Statistics Med* **10**: 1931–1941

Ando Y, Sugiura S, Ando M, Minami H, Nomura F, Sakai S and Shimokata K (1996) Acceptability of patients with brain metastases for clinical trials of chemotherapy for metastatic non-small-cell lung cancer. *Am J Clin Oncol* **19**: 478–482

Auchter RM, Lamond JP, Alexander E, Buatti JM, Chappell R, Friedman WA, Kinsella TJ, Levin AB, Noyes WR, Schultz CJ, Loeffler JS and Mehta MP (1996) A multi-institutional outcome and prognostic factor analysis of radiosurgery for resectable single brain metastasis [see comments]. *Int J Radiat Oncol Biol Phys* **35**: 27–35

Brechot JM, Chevret S, Nataf J, Le Gall C, Fretault J, Rochemaure J and Chastang C (1997) Diagnostic and prognostic value of Cyfra 21–1 compared with other tumour markers in patients with non-small cell lung cancer: a prospective study of 116 patients. *Eur J Cancer* **33**: 385–391

Buttner T, Lack B, Jager M, Wunsche W, Kuhn W, Muller T, Przuntek H and Postert T (1999) Serum levels of neuron-specific enolase and s-100 protein after single tonic-clonic seizures. *J Neurol* **246**: 459–461

Charloux A, Hedelin G, Dietemann A, Ifoundza T, Roeslin N, Pauli G and Quoix E (1997) Prognostic value of histology in patients with non-small cell lung cancer. *Lung Cancer* **17**: 123–134

Cooper EH and Splinter TAW (1987) Neuron specific enolase (NSE): a useful marker in small cell lung cancer. *Lung Cancer* **3**: 61–66

Cox DR (1972): Regression models and life-tables. *J R Stat Soc* **34B**: 187–220

Cunningham RT, Young IS, Winder J, O'Kane MJ, McKinstry S, Johnston CF, Dolan OM, Hawkins SA and Buchanan KD (1991) Serum neurone specific enolase (NSE) levels as an indicator of neuronal damage in patients with cerebral infarction. *Eur J Clin Invest* **21**: 497–500

Cunningham RT, Watt M, Winder J, McKinstry S, Lawson JT, Johnston CF, Hawkins SA and Buchanan KD (1996) Serum neurone-specific enolase as an indicator of stroke volume. *Eur J Clin Invest* **26**: 298–303

DeGiorgio CM, Correale JD, Gott PS, Ginsburg DL, Bracht KA, Smith T, Boutros R, Loskota WJ and Rabinowicz AL (1995) Serum neuron-specific enolase in human status epilepticus [see comments]. *Neurology* **45**: 1134–1137

DeGiorgio CM, Heck CN, Rabinowicz AL, Gott PS, Smith T and Correale J (1999) Serum neuron-specific enolase in the major subtypes of status epilepticus. *Neurology* **52**: 746–749

Depierre A, M.B, Moro D, Chevret S, Braun D, Quoix E, Lebeau B, Bronton JL, Lemarié E, Gouva S, Paillot N, Bréchot JM, Janicot H, Lebas FX, Terrious P and Chastahg C (1999) Phase II trial of neo-adjuvant chemotherapy in resectable stage I (except T1N0), II, IIIa non-small-cell lung cancer (NSCLC): the French experience. *Proc Am Soc Clin Oncol* **18**: 465a (A1792). 8

Diener-West M, Dobbins TW, Phillips TL and Nelson DF (1989) Identification of an optimal subgroup for treatment evaluation of patients with brain metastases using RTOG study 7916. *Int J Radiat Oncol Biol Phys* **16**: 669–673

Ellis R, Gregor A (1998) The treatment of brain metastases from lung cancer. *Lung Cancer* **20**: 81–84

Fogel W, Krieger D, Veith M, Adams HP, Hund E, Storch-Hagenlocher B, Buggle F, Mathias D and Hacke W (1997) Serum neuron-specific enolase as early predictor of outcome after cardiac arrest. *Crit Care Med* **25**: 1133–1138

Furuse K, Fukuoka M, Kawahara M, Nishikawa H, Takada Y, Kudoh S, Katagami N and Ariyoshi Y (1999) Phase III study of concurrent versus sequential thoracic radiotherapy in combination with mitomycin, vindesine, and cisplatin in unresectable stage III non-small-cell lung cancer. *J Clin Oncol* **17**: 2692–2699

 *© 2001 Cancer Research Campaign*

Gaspar L, Scott C, Rotman M, Asbell S, Phillips T, Wasserman T, McKenna WG and Byhardt R (1997) Recursive partitioning analysis (RPA) of prognostic factors in three Radiation Therapy Oncology Group (RTOG) brain metastases trials. *Int J Radiat Oncol Biol Phys* **37**: 745–751

Gould VE, Linnoila RI, Memoli VA and Warren WH (1983) Biology of disease: neuroendocrine components of the bronchopulmonary tract: hyperplasias, dysplasias, and neoplasms. *Lab Invest* **49**: 519–537

Harrell FE, Lee KL, Matchar DB, and Reichert TA (1985). Regression models for prognostic prediction: advantages, problems, and suggested solutions. *Cancer Treat Rep* **69**: 1071–1077

Hsiung CY, Leung SW, Wang CJ, Lo SK, Chen HC, Sun LM and Fang FM (1998) The prognostic factors of lung cancer patients with brain metastases treated with radiotherapy. *J Neurooncol* **36**: 71–77

Jorgensen LG, Hansen HH and Cooper EH (1989) Neuron specific enolase, carcinoembryonic antigen and lactate dehydrogenase as indicators of disease activity in small cell lung cancer. *Eur J Cancer Clin Oncol* **25**: 123–128

Kaplan EL and Meier P. (1958). Nonparametric estimation from incomplete observations. *J. Am. Stat. Assoc*. **53**: 457–481

Kelly K and Bunn PA, Jr (1998) Is it time to reevaluate our approach to the treatment of brain metastases in patients with non-small cell lung cancer? *Lung Cancer* **20**: 85–91

Komaki R, Pajak TF, Byhardt RW, Emami B, Asbell SO, Roach M, 3rd, Pedersen JE, Curran WJ, Jr, Lattin P and Russell AH (1993) Analysis of early and late deaths on RTOG non-small cell carcinoma of the lung trials: comparison with CALGB 8433. *Lung Cancer* **10**: 189–197

Komarnicky LT, Phillips TL, Martz K, Asbell S, Isaacson S and Urtasun R (1991) A randomized phase III protocol for the evaluation of misonidazole combined with radiation in the treatment of patients with brain metastases (RTOG-7916). *Int J Radiat Oncol Biol Phys* **20**: 53–58

Lagerwaard FJ, Levendag PC, Nowak PJ, Eijkenboom WM, Hanssens PE and Schmitz PI (1999) Identification of prognostic factors in patients with brain metastases: a review of 1292 patients. *Int J Radiat Oncol Biol Phys* **43**: 795–803

Lonjon M, Paquis P, Michiels JF, Frenay M, Bensadoun RJ, Chatel M, Roche JL and Grellier P (1994) [Single cerebral metastasis of bronchopulmonary cancers]. *Rev Neurol* **150**: 216–221

Martens P, Raabe A and Johnsson P (1998) Serum S-100 and neuron-specific enolase for prediction of regaining consciousness after global cerebral ischemia. *Stroke* **29**: 2363–2366

Merrill RM, Henson DE and Barnes M (1999) Conditional survival among patients with carcinoma of the lung [see comments]. *Chest* **116**: 697–703

Missler U, Wiesmann M, Friedrich C and Kaps M (1997) S-100 protein and neuron-specific enolase concentrations in blood as indicators of infarction volume and prognosis in acute ischemic stroke [see comments]. *Stroke* **28**: 1956–1960

Mountain CF (1997) Revisions in the international item for staging lung cancer. *Chest* **111**: 1710–1717

Newman SJ and Hansen HH (1974) Proceedings: Frequency, diagnosis, and treatment of brain metastases in 247 consecutive patients with bronchogenic carcinoma. *Cancer* **33**: 492–496

Nguyen LN, Maor MH and Oswald MJ (1998) Brain metastases as the only manifestation of undetected primary tumor. *Cancer* **83**: 2181–2184

Nicolson GL (1987) Tumor cell instability, diversification, and progression to the metastatic phenotype: from oncogene to oncofetal expression. *Cancer Res* **47**: 1473–1487

Nussbaum ES, Djalilian HR, Cho KH and Hall WA (1996) Brain metastases. Histology, multiplicity, surgery, and survival. *Cancer* **78**: 1781–1788

Paesmans M, Sculier JP, Libert P, Bureau G, Dabouis G, Thiriaux J, Michel J, Van Cutsem O, Sergysels R and Mommen P (1995) Prognostic factors for survival in advanced non-small-cell lung cancer: univariate and multivariate analyses including recursive partitioning and amalgamation algorithms in 1,052 patients. The European Lung Cancer Working Party. *J Clin Oncol* **13**: 1221–1230

Paesmans M, Sculier JP, Libert P, Bureau G, Dabouis G, Thiriaux J, Michel J, Van Cutsem O, Sergysels R, Mommen P and Klastersky J (1997) Response to chemotherapy has predictive value for further survival of patients with advanced non-small cell lung cancer: 10 years experience of the European Lung Cancer Working Party. *Eur J Cancer* **33**: 2326–2332

Postmus PE and Smit EF (1999) Chemotherapy for brain metastases of lung cancer: a review. *Ann Oncol* **10**: 753–759

Pujol JL, Grenier J, Daures JP, Daver A, Pujol H and Michel FB (1993) Serum fragment of cytokeratin subunit 19 measured by CYFRA 21–1 immunoradiometric assay as a marker of lung cancer. *Cancer Res* **53**: 61–66

Radice PA, Matthews MJ, Ihde DC, Gazdar AF, Carney DN, Bunn PA, Cohen MH, Fossieck BE, Makuch RW and Minna JD (1982) The clinical behavior of “mixed” small cell/large cell bronchogenic carcinoma compared to “pure” small cell subtypes. *Cancer* **50**: 2894–2902

Ryan GF, Ball DL and Smith JG (1995) Treatment of brain metastases from primary lung cancer. *Int J Radiat Oncol Biol Phys* **31**: 273–278

Schoerkhuber W, Kittler H, Sterz F, Behringer W, Holzer M, Frossard M, Spitzauer S and Laggner AN (1999) Time course of serum neuron-specific enolase. A predictor of neurological outcome in patients resuscitated from cardiac arrest. *Stroke* **30**: 1598–1603

Sen M, Demiral AS, Cetingoz R, Alanyali H, Akman F, Senturk D and Kinary M (1998) Prognostic factors in lung cancer with brain metastasis. *Radiother Oncol* **46**: 33–38

Shepherd FA (1999) Chemotherapy for non-small cell lung cancer: have we reached a new plateau? *Semin Oncol* **26**: 3–11

Simon R and Altman DG (1994). Statistical aspects of prognostic factor studies in oncology. *Br J Cancer* **69**: 979–985

Sobin LH, Hermanek P and Hutter RVP (1987) TNM classification of malignant tumours. 4th edition. UICC: Geneva

Sorensen JB, Hansen HH, Hansen M and Dombernowsky P (1988) Brain metastases in adenocarcinoma of the lung: frequency, risk groups, and prognosis. *J Clin Oncol* **6**: 1474–1480

Swift PS, Phillips T, Martz K, Wara W, Mohiuddin M, Chang CH and Asbell SO (1993) CT characteristics of patients with brain metastases treated in RTOG study 79–16. *Int J Radiat Oncol Biol Phys* **25**: 209–214

Tisi GM, F.P., Peters RM, Pearson G, Carr D, Lee RE and Selawry O (1982) American Thoracic Society: clinical staging of primary lung cancer. *Am Rev Respir Dis* **125**: 659–664

Wieskopf B, Demangeat C, Purohit A, Stenger R, Gries P, Kreisman H and Quoix E (1995) Cyfra 21–1 as a biologic marker of non-small cell lung cancer. Evaluation of sensitivity, specificity, and prognostic role [see comments]. *Chest* **108**: 163–169

World Health Organization (1982) The World Health Organization histological typing of the lung tumors. 2nd Ed. *Am J Clin Pathol* **77**: 123–136

Wunderlich MT, Ebert AD, Kratz T, Goertler M, Jost S and Herrmann M (1999) Early neurobehavioral outcome after stroke is related to release of neurobiochemical markers of brain damage. *Stroke* **30**: 1190–1195

Yesner R and Carter D (1982) Pathology of carcinoma of the lung. Changing patterns. *Clin Chest Med* **3**: 257–289

Zimm S, Wampler GL, Stablein D, Hazra T and Young HF (1981) Intracerebral metastases in solid-tumor patients: natural history and results of treatment. *Cancer* **48**: 384–394

Zubrod CG, Schneiderman M and Frei E Jr (1960) Appraisal of methods for the study of chemotherapy of cancer in man: Comparative therapeutic trial of nitrogen mustard and triethylene thiophosphoramide. *J Chron Dis* **11**: 7–33

*© 2001 Cancer Research Campaign*
